# Purification, crystallization and preliminary X-ray diffraction analysis of a soluble variant of the monoglyceride lipase Yju3p from the yeast *Saccharomyces cerevisiae*

**DOI:** 10.1107/S2053230X15001557

**Published:** 2015-01-28

**Authors:** Srinivasan Rengachari, Philipp Aschauer, Christian Sturm, Monika Oberer

**Affiliations:** a Institute of Molecular Biology, University of Graz, Humboldtstrasse 50/3, 8010 Graz, Austria

**Keywords:** s-Yju3p, monoacylglycerol lipase, monoglyceride lipase

## Abstract

The protein Yju3p is the orthologue of monoglyceride lipases in the yeast *Saccharomyces cerevisiae*. A soluble variant of this lipase termed s-Yju3p (38.3 kDa) was generated and purified to homogeneity by affinity and size-exclusion chromatography. s-Yju3p was crystallized in a vapour-diffusion setup at 293 K and a complete data set was collected to 2.4 Å resolution. The crystal form was orthorhombic (space group *P*2_1_2_1_2_1_), with unit-cell parameters *a* = 77.2, *b* = 108.6, *c* = 167.7 Å. The asymmetric unit contained four molecules with a solvent content of 46.4%.

## Abstract 2

A soluble variant of the monoglyceride lipase Yju3p was successfully expressed, purified and crystallized. Diffraction data were collected to 2.4 Å resolution.

## 1. Introduction   

Lipases are enzymes that break down lipids by catalyzing the hydrolysis of ester bonds. Monoglyceride lipases (MGLs) specifically catalyze the breakdown of monoglycerides (MGs) into molecules of fatty acids and glycerol and have been identified in all kingdoms of life. They are involved in the digestive uptake and metabolism of nutritional lipids and in the synthesis and remodelling of membrane lipids. In intracellular lipolysis, stored triglycerides are mobilized from lipid droplets through the consecutive action of different lipases, where MGLs catalyze the last step (Lass *et al.*, 2011 ▶). MGLs have high substrate specificity for MG, but have low stereoselectivity (Tornqvist & Belfrage, 1976 ▶; Heier *et al.*, 2010 ▶; Imamura & Kitaura, 2000 ▶; Navia-Paldanius *et al.*, 2012 ▶). MGLs have been shown to be involved in the endocannabinoid metabolism in the mammalian brain and also aid in bacterial cellular defence (Batovska *et al.*, 2009 ▶; Dinh *et al.*, 2004 ▶; Conley & Kabara, 1973 ▶; Kabara *et al.*, 1978 ▶; Isaacs, 2001 ▶; Preuss *et al.*, 2005 ▶).

Despite their ubiquitous expression and important physiological functions, only the three-dimensional structures of MGLs from *Homo sapiens* and *Bacillus* sp. H257 have been determined to date (Bertrand *et al.*, 2010 ▶; Labar *et al.*, 2010 ▶; Schalk-Hihi *et al.*, 2011 ▶; Rengachari *et al.*, 2012 ▶, 2013 ▶). The structures revealed an α/β hydrolase fold with a dynamic yet topologically conserved cap region. The amphiphilic nature of this cap region in eukaryotes might be necessary for the interaction with the lipid–water interface in order to extract MGs from lipid membranes (Schalk-Hihi *et al.*, 2011 ▶).

This study focuses on Yju3p, the *Saccharomyces cerevisiae* orthologue of mammalian MGL (Heier *et al.*, 2010 ▶). Studies using mass spectroscopy have shown that Yju3p binds to lipid droplets and is not found in cytosolic cell fractions (Athenstaedt *et al.*, 1999 ▶; Athenstaedt & Daum, 2005 ▶). These findings imply that triglycerides stored in lipid droplets can be completely degraded to free fatty acids and glycerol on the lipid droplet surface. In this study, we report the crystallization of a soluble variant of the *S. cerevisiae* MGL, s-Yju3p, in an orthorhombic space group and preliminary diffraction analysis at 2.6 Å resolution, and describe the data quality and other properties of the crystal.

## 2. Materials and methods   

### 2.1. Macromolecule production   

Overexpression of Yju3p in *Escherichia coli* did not result in sufficient soluble target protein after cell lysis without adding detergents. A soluble variant of Yju3p was generated from the previously described construct by employing site-directed mutagenesis (Heier *et al.*, 2010 ▶). The details and the rationale for this solubility-enhancement mutation L175S will be described elsewhere. The soluble variant containing this mutation will be referred to as s-Yju3p.

*E. coli* BL21 (DE3) cells harbouring a pProExHtb vector (LifeTechnologies) subcloned with s-Yju3p were grown in LB (Luria–Miller) broth (Carl Roth GmbH, Karlsruhe, Germany) at 37°C from an overnight seed culture until they reached an optical density (OD_600_) of 0.7. Gene expression was induced using 1 m*M* IPTG at 37°C for 4 h. The cells were harvested and lyzed by sonication in lysis buffer (20 m*M* Tris–HCl pH 8.0, 100 m*M* NaCl). The lysate was centrifuged at 22 000*g* for 30 min and the soluble fraction was loaded onto an Ni–NTA agarose resin column (Qiagen, Hilden, Germany). The protein was eluted with 20 m*M* Tris–HCl pH 8.0, 100 m*M* NaCl, 250 m*M* imidazole, 5% glycerol and was dialyzed against buffer *A* (20 m*M* Tris–HCl pH 8.0, 100 m*M* NaCl, 5% glycerol, 1 m*M* EDTA, 1 m*M* DTT). Subsequently, the protein was concentrated and loaded onto a Superdex 200 column (GE Healthcare) in buffer *A* at a flow rate of 2 ml min^−1^. The purity of the protein was examined using SDS–PAGE and the protein concentration was determined by UV spectroscopy using an extinction coefficient of 44 810 *M* ^−1^ cm^−1^. Macromolecule-production information is summarized in Table 1 ▶.

### 2.2. Crystallization   

Initial crystallization trials were performed with a 16.4 mg ml^−1^ s-Yju3p solution using the sitting-drop vapour-diffusion method with a 1:1 ratio of protein and reservoir solution (0.5 µl each). Initial crystals were obtained from the Morpheus screen (Molecular Dimensions, Suffolk, England) in a drop consisting of 0.1 *M* MOPS/HEPES-Na pH 7.5, 10%(*w*/*v*) PEG 20 000, 20%(*v*/*v*) PEG MME 550, 0.03 *M* sodium nitrate, 0.03 *M* disodium hydrogen phosphate, 0.03 *M* ammonium sulfate. This crystal was used to prepare a micro-seeding stock (D’Arcy *et al.*, 2007 ▶). The seeding stock was diluted 1:1000 and used to set up the Morpheus screen again with a drop ratio of 0.4:0.4:0.2 µl protein solution (14 mg ml^−1^), reservoir solution and seeding stock, respectively. A crystal diffracting to 9 Å resolution was obtained from a drop consisting of 0.1 *M* bicine/Trizma base pH 8.5, 10%(*w*/*v*) PEG 20 000, 20%(*v*/*v*) PEG MME 550, 0.03 *M* sodium nitrate, 0.03 *M* disodium hydrogen phosphate, 0.03 *M* ammonium sulfate. Upon further optimization using the hanging-drop method, a crystal diffracting to 2.4 Å resolution was obtained from a drop containing the same conditions except that the pH of the bicine/Trizma buffer stock was 8.7. A 5 µl drop of 2:2:1 ratio of protein solution (14 mg ml^−1^), reservoir solution and seeding stock (1:100), respectively, was used for a second optimization using the hanging-drop method with the same conditions (Table 2 ▶). All crystallization experiments were performed at a temperature of 293 K.

### 2.3. Data collection and processing   

Diffraction data for the s-Yju3p crystals were collected on the PXIII beamline at Swiss Light Source (SLS), Villigen, Switzerland. The 270-image data set (oscillation angle 1°) was processed by *XDS* and scaled using *AIMLESS* (Kabsch, 2010 ▶; Evans & Murshudov, 2013 ▶). Details of the data collection and processing and statistics describing the quality of the data are listed in Table 3 ▶.

## 3. Results and discussion   

**Figure 1:**
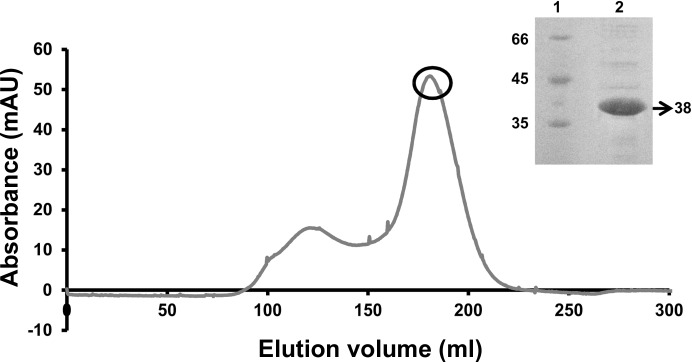
Size-exclusion chromatography and SDS–PAGE analysis of s-Yju3p. The purified and monomeric fraction of s-Yju3p from the size-exclusion column was used for crystallization trials.

**Figure 2:**
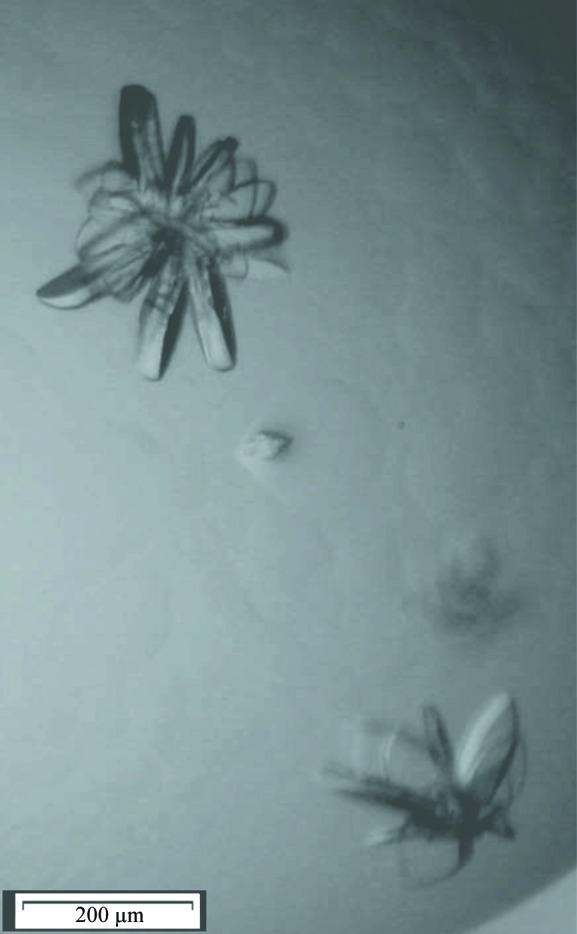
Crystals of s-Yju3p obtained by the hanging-drop method at 293 K in 0.1 *M* bicine/Trizma base pH 8.7, 10%(*w*/*v*) PEG 20 000, 20%(*v*/*v*) PEG MME 550, 0.03 *M* sodium nitrate, 0.03 *M* disodium hydrogen phosphate, 0.03 *M* ammonium sulfate. The scale bar is 200 µm in length.

**Figure 3:**
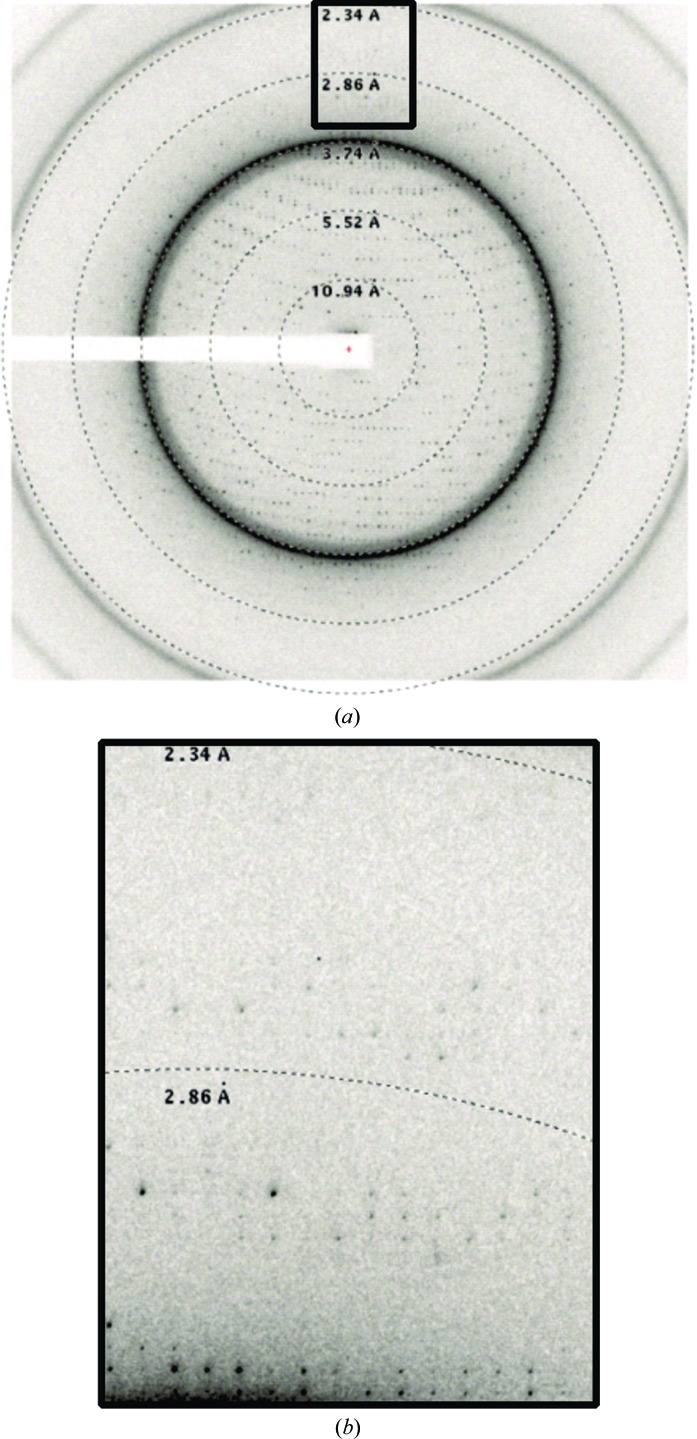
X-ray diffraction image of s-Yju3p; the inset shows a close-up of high-resolution spots extending to 2.4 Å.

s-Yju3p was purified to apparent homogeneity by metal-affinity chromatography followed by size-exclusion chromatography. The major elution volume peak corresponds to monomeric s-Yju3p with an apparent molcular weight of 38 kDa (Fig. 1 ▶).

s-Yju3p crystallized as a cluster of platelets and crystals were detected after 7 days (Fig. 2 ▶). Single crystals from the cluster were harvested and flash-cooled in liquid nitrogen without the addition of further cryoprotectants (the addition of glycerol as a cryoprotectant led to the disintegration of the crystals). 270 diffraction images (oscillation angle 1°) were collected at 100 K from the best crystal diffracting to 2.4 Å resolution (Fig. 3 ▶), but the data set was cut at 2.6 Å during scaling based on the CC_1/2_ and *R*_merge_ values. The data set was integrated in the primitive orthorhombic space group *P*2_1_2_1_2_1_ as verified by *POINTLESS* (Evans, 2006 ▶). The unit-cell parameters were *a* = 77.2, *b* = 108.6, *c* = 167.7 Å. According to the Matthews coefficient, the asymmetric unit contained four molecules with 46.4% solvent content [2.29 Å^3^ Da^−1^, *P*(tot) = 0.70; Matthews, 1968 ▶]. *Phenix.xtriage* revealed that the crystal form contained neither twinning nor pseudo-translational symmetry (Adams *et al.*, 2010 ▶). Attempts to derive the phases of s-Yju3p by molecular replacement failed owing to a lack of high-identity structures. According to a *BLAST* search, the protein of known structure that has the highest sequence identity is MGL from *H. sapiens*, with a sequence identity of 24% (based on 97% query coverage). Hence, approaches leading to experimental phase determination are being pursued, *i.e.* the use of selenomethionine-incorporated protein for SAD and the use of heavy-atom soaking for MIR/MAD.

MGLs are a relevant class of enzymes which have been heavily studied with respect to their biochemical and pharmacological properties for industrial applications. Our understanding of the structural restraints determining the enzyme specificity is still vague, yet will aid our knowledge of substrate specificity. The structure of Yju3p will thus pave the way for a deeper understanding of the structure–function relationship of MGLs.

## Figures and Tables

**Table 1:** Macromolecule-production information

Source organism	*S. cerevisiae*
DNA source	pYEX-4T-1; insert cut out using BamHI and EcoRI
Cloning vector	pET21a; insert cut out using BamHI and XhoI
Expression vector	pProExHtb
Expression host	*E. coli*
Complete amino-acid sequence of the construct produced	MSYYHHHHHHDYDIPTTENLYFQGAMGSAPYPYKVQTTVPELQYENFDGAKFGYMFWPVQNGTNEVRGRVLLIHGFGEYTKIQFRLMDHLSLNGYESFTFDQRGAGVTSPGRSKGVTDEYHVFNDLEHFVEKNLSECKAKGIPLFMWGHSMGGGICLNYACQGKHKNEISGYIGSGPLIILHPHTMYNKPTQIIAPLLAKFSPRVRIDTGLDLKGITSDKAYRAFLGSDPMSVPLYGSFRQIHDFMQRGAKLYKNENNYIQKNFAKDKPVIIMHGQDDTINDPKGSEKFIRDCPSADKELKLYPGARHSIFSLETDKVFNTVFNDMKQWLDKHTTTEAKP

**Table 2:** Crystallization

Method	Vapour diffusion: sitting/hanging-drop method
Plate type	Linbro (24-well)
Temperature (K)	293
Protein concentration (mgml^1^)	14
Buffer composition of protein solution	20m*M* TrisHCl pH 8.0, 100m*M* NaCl, 5% glycerol, 1m*M* EDTA, 1m*M* DTT
Composition of reservoir solution	0.1*M* bicine/Trizma base pH 8.7, 10%(*w*/*v*) PEG 20 000, 20%(*v*/*v*) PEG MME 550, 0.03*M* sodium nitrate, 0.03*M* disodium hydrogen phosphate, 0.03*M* ammonium sulfate
Volume and ratio of drop	5l; 2:2:1 ratio of protein, reservoir solution and seeding stock
Volume of reservoir (ml)	0.5

**Table 3:** Data collection and processing Values in parentheses are for the highest resolution shell.

Diffraction source	PXIII beamline, SLS
Wavelength ()	0.999900
Temperature (K)	100
Detector	MAR 225 CCD
Crystal-to-detector distance (mm)	250
Rotation range per image ()	1.00
Total rotation range ()	270
Space group	*P*2_1_2_1_2_1_
*a*, *b*, *c* ()	77.2, 108.6, 167.7
, , ()	90, 90, 90
Mosaicity ()	0.10
Resolution range ()	45.272.60 (2.702.60)
Total No. of reflections	391577
No. of unique reflections	43595
Completeness (%)	99.1 (98.9)
Multiplicity	9.0 (9.1)
*I*/(*I*)	11.6 (2.5)
*R*_meas_	0.191 (1.116)
*R*_p.i.m._	0.063 (0.365)
CC_1/2_	0.992 (0.712)
Overall *B* factor from Wilson plot (^2^)	30.5
